# Nepal’s vulnerability to Nipah virus transmission: an urgent call for preparedness

**DOI:** 10.1093/sumbio/qvag015

**Published:** 2026-04-25

**Authors:** Bhupendra Lama, Uday Narayan Yadav, Gopiram Syangtan, Alaka Bhandari, Binod GC, Mukesh Rai, Martyn D Kirk, Binod Rayamajhee

**Affiliations:** Department of Microbiology, Tribhuvan University, Amrit Campus, 44600, Kathmandu, Nepal; National Institute of Bio Sciences and Research Centre, 44600, Kathmandu, Nepal; National Centre for Epidemiology and Population Health, Australian National University (ANU), Acton ACT 2601, Canberra, Australia; International Centre for Future Health Systems, UNSW, 2033 NSW, Australia; Centre for Research, Policy and Implementation, 56613, Biratnagar, Nepal; Department of Microbiology, Tribhuvan University, Amrit Campus, 44600, Kathmandu, Nepal; National Institute of Bio Sciences and Research Centre, 44600, Kathmandu, Nepal; Department of Infection and Immunology, Kathmandu Research Institute for Biological Sciences (KRIBS), 44700, Kathmandu, Nepal; Susan Wakil School of Nursing, Faculty of Medicine and Health, University of Sydney, 2050 NSW, Australia; Department of Infection and Immunology, Kathmandu Research Institute for Biological Sciences (KRIBS), 44700, Kathmandu, Nepal; Department of Biomedical Sciences, Cedars-Sinai Medical Center, Los Angeles, CA 90048, United States; Department of Environmental Science, Baylor University, Waco, TX 76706, United States; National Centre for Epidemiology and Population Health, Australian National University (ANU), Acton ACT 2601, Canberra, Australia; Department of Infection and Immunology, Kathmandu Research Institute for Biological Sciences (KRIBS), 44700, Kathmandu, Nepal; Faculty of Medicine, Health and Human Sciences, Macquarie University, 2109 NSW, Australia; School of Optometry and Vision Science, Faculty of Medicine, UNSW, 2033 NSW, Australia

**Keywords:** Nipah virus, Nepal, zoonotic spillover, One Health approach, disease surveillance, outbreak preparedness

## Abstract

The confirmation of two Nipah virus (NiV) infections among healthcare workers in West Bengal, India, in December 2025 highlights the ongoing regional threat of this highly contagious zoonotic pathogen and the risk of nosocomial transmission before clinical suspicion. Increasing reports of human-to-human transmission in South Asia call for stronger preparedness efforts in neighboring countries. Nepal is particularly vulnerable due to its open border with India, high population mobility, ecological suitability for *Petropus* bat reservoirs, mixed livestock farming, limited surveillance, and diagnostic capacity. The absence of reported cases likely reflects underdetection rather than the absence of risk. Current gaps include weak infection prevention and control, limited laboratory readiness, minimal wildlife and livestock surveillance, and inadequate cross-border coordination. We propose priority actions based on the One Health approach, including bat roost mapping, cross-border information sharing, targeted community risk communication, strengthening tertiary hospitals and laboratory capacity, enhanced veterinary surveillance, and the development of a national outbreak preparedness and response plan for future potential NiV or other emerging pathogen outbreaks.

**Sustainability Statement**

This commentary aligns with US SDG 3 (Good Health and Well-being) by promoting improved surveillance, early detection, and infection prevention capacity for NiV, with measurable outcomes including increased case detection rates, reduced time to diagnosis, and strengthened healthcare preparedness. It contributes to SDG 13 (Climate Action) by integrating surveillance and preparedness strategies with environmental monitoring of bat habitats, supporting adaptive responses to ecological changes influencing zoonotic spillover risk. The proposed One Health approach also advances SDG 17 (Partnerships for the Goals) through strengthened cross-border collaboration, multisectoral coordination, and data sharing, with measurable indicators such as the establishment of joint surveillance mechanisms and coordinated outbreak response frameworks in the region.

## Introduction

The confirmation of two cases of Nipah virus infections in India’s West Bengal state at the end of December 2025 has once again drawn regional and international attention to this deadly zoonotic pathogen (Shah et al. 2026, WHO 2026). Notably, both cases involved healthcare workers, an alarming signal for potential nosocomial transmission of NiV, particularly before clinical suspicion is established, highlighting the importance of rapid diagnosis, early triage, and adequate infection prevention and control (IPC) measures. Unlike earlier outbreaks in Malaysia, Singapore, and the Philippines, where transmission was primarily animal-to-human, recent South Asian clusters increasingly reflect sustained human-to-human transmission (Khan et al. 2024). The rapid introduction of airport screening measures in Thailand, Malaysia, Singapore, and Hong Kong underscores the transboundary threat and the speed at which NiV concerns mobilize international public health responses. For Nepal, the COVID-19 pandemic demonstrated how delays in pathogen detection and weak public health infrastructure can magnify epidemic spread, highlighting the urgent need to strengthen surveillance, reporting, and rapid-response capacity in a resource-constrained healthcare system (Rayamajhee et al. 2021).

NiV remains one of the most lethal zoonotic pathogens, with fruit bats/Indian flying fox (*Pteropus* spp.) as the natural reservoirs, and pigs and livestock acting as amplifying hosts (Epstein et al. 2020, Li et al. 2023). The case-fatality rates in humans typically range from 40% to 80% and exceeding 90% in some outbreaks (Hauser et al. 2021). With no licensed vaccines or specific therapeutics, the pathogen’s epidemic potential is widely acknowledged; therefore, the WHO lists NiV as one of the highest-priority pathogens for pandemic preparedness (Mohapatra et al. 2024).

**Figure 1:**
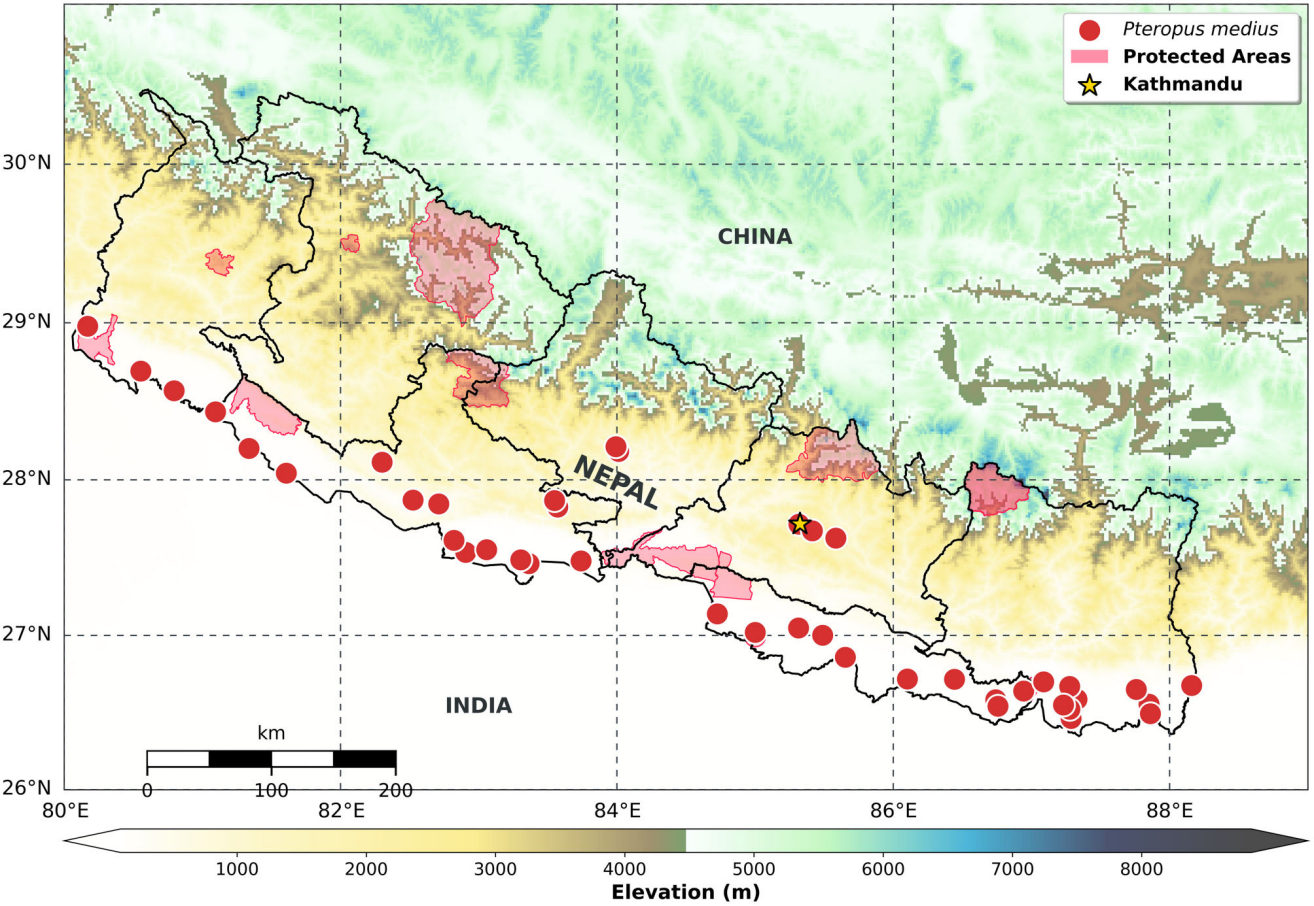
Distribution of *Pteropus medius* bat colonies across Nepal, highlighting potential hotspots for NiV spillover. Roosting colonies of *P. medius* have been documented in Kathmandu, Nepal since the 1960s, with current distributions spanning the Tarai, Inner Tarai, and central Middle Mountain regions across elevations from 75 m to 1322 m above sea level. Most recorded colonies occur outside protected areas. The map was generated using Python, the elevation data were obtained from NOAA (www.ngdc.noaa.gov/mgg/global/relief/ETOPO1/tiled), and the protected areas were downloaded from protected-areas-in-nepal-uji.hub.arcgis.com. .

For Nepal, the recent West Bengal event is an urgent warning about the potential for a widespread outbreak of NiV. Nepal shares an open border with India, alongside dense mobility corridors, and cross-border active livestock and goods trade can introduce and spread pathogens in Nepal (Joshi et al. 2023). The absence of documented human cases in Nepal cannot be interpreted as evidence of no risk, particularly in a context where wildlife surveillance, livestock monitoring, and diagnostic capacity remain limited, as the evidence from South Asian countries indicating that NiV transmission may remain undetected without robust surveillance systems (Luby et al. 2009, Epstein et al. 2020, Naidoo et al. 2026). Ecological suitability further heightens concern as *Pteropus* bat roosts are widely distributed across Nepal, including districts bordering eastern and northeastern states of India (Fig. 1) (Joshi et al. 2023). In 2020, evidence from Bangladesh reported continuous viral shedding in bats and fruit-mediated spillover to humans (Epstein et al. 2020). Recent surveillance in India’s eastern border states, adjacent to Nepal, detected NiV RNA and seropositivity in *P. medius*, confirming viral circulation beyond historically recognized hotspots (Mohandas et al. 2024).

Nipah outbreaks remain a significant and ongoing public health risk, underscoring the need for strengthened preparedness to minimize their potential impact (Asokan et al. 2026). Nepal’s ecological, cultural, and agricultural determinants collectively compound the risk of NiV exposure. Seasonal bat migration, consumption of bats as bushmeat by several Indigenous communities, and widespread ingestion of bat–contaminated fruits create direct human-bat interfaces (Joshi et al. 2023). Moreover, mixed livestock-crop farming, pig rearing near bat roosts, and minimal biosecurity facilitate cross–species transmission of NiV in Nepal (Epstein et al. 2020, Li et al. 2023). Furthermore, veterinary surveillance for NiV in livestock remains limited in Nepal, representing a critical gap in the country’s prevention and early detection capacity (Yadav et al. 2025).

Experience from repeated outbreaks in neighboring India and Bangladesh demonstrates that NiV exploits vulnerabilities in IPC and gaps in clinical preparedness. Secondary transmission commonly occurs before NiV is suspected, placing families and healthcare workers at extreme risk of fatal infections (Hauser et al. 2021, Joshi et al. 2023). Nepal currently lacks the surge capacity to rapidly contain NiV, with inconsistent IPC protocols, fragmented triage, and uneven availability of personal protective equipment (PPE) and isolation facilities, limited biosafety level (BSL)-compatible diagnostic capacity, very limited cross-border screening, and virtually no systematic NiV surveillance in bats, livestock, or suspected febrile neurological illness patients. These diagnostic blind spots heighten the risk of silent viral circulation across the country (Joshi et al. 2023, Mohandas et al. 2024).

Despite increasing recognition of the importance of a one health approach, its implementation in Nepal remains constrained by structural and resource limitations (Dahal et al. 2017, Acharya et al. 2019). Existing surveillance systems are fragmented across human, animal, and environmental health sectors, with limited integration and data sharing (Napit et al. 2025). Laboratory capacity for high-consequence pathogens is restricted, and there is a shortage of trained personnel in specialized diagnostics and outbreak response (Cote et al. 2025). In addition, financial and logistical constraints, coupled with competing public health priorities, pose challenges to sustained investment in emerging infectious disease preparedness (Khanal et al. 2025, Mehta et al. 2026). Strengthening political commitment and institutional coordination will therefore be critical to operationalizing NiV preparedness strategies in resource-scarce countries such as Nepal (Sharma et al. 2018).

Preparedness must shift from a reactive to a pre–emptive approach, supported by generic frameworks for early detection and mitigation of transmission of emerging infectious diseases, including high-consequence zoonotic pathogens such as NiV. We therefore propose the following five priority actions to prevent and control potential future NiV outbreaks in Nepal and other low- and middle-income countries in the region:

1. Systematic mapping of bat roosts and spillover hotspots using non-invasive, one health surveillance system that prioritizes habitat conservation, interface-focused monitoring, and community engagement, reducing zoonotic risk without disrupting the essential ecological roles of bats.
2. Cross-border surveillance and rapid information sharing with Indian and other neighboring country authorities to enable early outbreak detection through coordinated multisectoral participation of ecologists, public health professionals, veterinary services, and local communities.
3. Targeted risk communication and community awareness programs for high-risk populations, emphasizing specific, evidence-informed precautionary measures (e.g. avoiding half-eaten/contaminated fruits and bat consumption), recognizing that current Indian cases have not involved foodborne transmission but that these pathways are well-documented elsewhere in South Asia.
4. Strengthening selected tertiary hospitals and laboratories for preparedness and response to emerging high-consequence infectious diseases, with emphasis on robust IPC, rapid isolation capability, safe sample collection and transport, and clear referral pathways to regional BSL-4 reference laboratories when required.
5. Enhanced veterinary and wildlife surveillance, particularly in pig farms and livestock markets.
6. A national NiV preparedness and response plan, aligned with One Health principles, enabling coordinated action across human, animal, and environmental health sectors.

Although these recommendations align with established One Health strategies in South Asia, their implementation in Nepal requires context-specific adaptation. Among the six proposed priority actions, community risk communication and awareness are immediate recommendations that are highly feasible to implement. Similarly, mapping of bat roosts and strengthening cross-border surveillance are also feasible within the existing surveillance systems of the country, although these would require expansion to achieve broader coverage (Shah et al. 2026). In contrast, strengthening tertiary hospitals and laboratory capacity, enhancing veterinary and wildlife surveillance, and developing a national Nipah virus preparedness and response plan represent longer-term, resource-intensive priorities (Yeasmin et al. 2025, Gupta et al. 2026). These actions require sustained investment, infrastructure development, and coordinated multisectoral collaboration across human, animal, and environmental health systems (Gupta et al. 2026). To mitigate the risk of future Nipah virus outbreaks in Nepal, several proposed actions can be implemented by leveraging existing systems and regional collaborations. Bat roost mapping can build upon ongoing ecological and wildlife surveillance initiatives within Nepal, facilitating the targeted identification of high-risk spillover zones (Bhattarai et al. 2025, Napit et al. 2025). Cross-border surveillance can utilize established public health coordination mechanisms between Nepal and India to enable timely information sharing and early outbreak detection. In addition, referral pathways to regional high-containment laboratories are feasible through existing international collaborations and WHO-supported laboratory networks, ensuring access to specialized diagnostic capacity when required (Cote et al. 2025). These context-specific considerations enhance the feasibility and relevance of the proposed actions within Nepal’s healthcare system. Furthermore, healthcare preparedness must prioritize strengthening triage systems to enable early identification and isolation of suspected cases, particularly in settings where clinical suspicion may be low during initial presentation. Standardized screening protocols, coupled with clear referral pathways, are essential to minimize delays in diagnosis and reduce the risk of nosocomial transmission (Joshi et al. 2023). In addition, protecting healthcare workers is equally important and requires consistent availability and appropriate use of PPE, regular training in IPC practices, and reinforcement of standard and transmission-based precautions (Joshi et al. 2023, Khanal et al. 2025). In endemic-risk regions, maintaining a high index of clinical suspicion for Nipah virus infection among patients presenting with acute febrile illness or associated symptoms is vital to ensure timely detection and response (Asokan et al. 2026, Gupta et al. 2026).

Importantly, to ensure the sustainability of the proposed interventions, these should incorporate local community participation and community health workers (CHWs)-led approaches to design context-specific and effective prevention strategies (Gupta and Sigdel 2024). The involvement of female community health volunteers (FCHVs) in dengue control awareness has been effective in Nepal; therefore, a similar approach could be adapted for NiV preparedness (Ghimire and Singh 2025). Active community engagement in risk communication, surveillance, and behavioral change interventions can enhance early detection and reduce spillover risk (Durrance-Bagale et al. 2025). In addition, integration of NiV preparedness within existing climate adaptation and environmental health programs can further strengthen resilience, particularly in regions affected by ecological changes influencing bat habitats (Gentle and Mainaly 2024).

In the absence of licensed vaccines or specific therapeutic interventions for NiV, preparedness efforts should focus on early detection, rapid response, and mitigation of transmission rather than complete outbreak control (Gupta et al. 2026, Haque et al. 2026). The proposed actions are therefore intended as a staged preparedness framework rather than standalone interventions, recognizing the practical constraints of implementation in resource-limited settings. Given Nepal’s ecological suitability, livestock interfaces, porous borders, and the recurrent emergence of NiV in neighboring regions, the country must recognize that it is *vulnerable and underprepared*. The West Bengal cluster should serve as an urgent call for investment in surveillance, diagnostics, and One Health-based preparedness before the next spillover event turns into a national health emergency.

## Data Availability

No new data were generated or analyzed in this study.

## References

[bib1] Acharya K P, Karki S, Shrestha K et al. One health approach in Nepal: scope, opportunities and challenges. One Health. 2019;8:100101. 10.1016/j.onehlt.2019.100101 31485475 PMC6715885

[bib2] Asokan S, Luke M S, Atiyah H M et al. Nipah virus as a pandemic threat: current knowledge, diagnostic gaps, and future research priorities. Diagn Microbiol Infect Dis. 2026;114:117141. 10.1016/j.diagmicrobio.2025.117141 41092535

[bib3] Bhattarai P K, Sharma B, Bhattarai B et al. Spatio-temporal colony dynamics and roost tree preferences of Indian flying fox, *Pteropus medius*, in Jhapa, Nepal. Biotropica. 2025;57:e70130 10.1111/btp.70130.

[bib4] Côté F L, Lahrichi N, Gralla E et al. Within-laboratory sars-cov-2 real time pcr testing operations in Nepal: a simulation-based analysis. Lancet Reg Health Southeast Asia. 2025;36:100584. 10.1016/j.lansea.2025.100584 40337599 PMC12056959

[bib5] Dahal R, Upadhyay A, Ewald B. One health in south asia and its challenges in implementation from stakeholder perspective. Vet Rec. 2017;181:626. 10.1136/vr.104189 29084821

[bib6] Durrance-Bagale A, Basnet H, Singh N B et al.‘Community people are the most powerful resources’: qualitative critical realist analysis and framework to support co-produced responses to zoonotic disease threats with(in) Nepali communities. BMC Public Health. 2025;25:1430. 10.1186/s12889-025-22657-9 40241058 PMC12001725

[bib7] Epstein J H, Anthony S J, Islam A et al. Nipah virus dynamics in bats and implications for spillover to humans. Proc Natl Acad Sci USA. 2020;117:29190–201. 10.1073/pnas.2000429117 33139552 PMC7682340

[bib8] Gentle P, Mainaly J. Commitment, actions, and challenges on locally led climate change adaptation in Nepal. Clim Risk Manag. 2024;46:100650 10.1016/j.crm.2024.100650.

[bib9] Ghimire S, Singh DR. A qualitative exploration of community stakeholders perspectives on dengue outbreak management in urban Nepal: navigational insights and challenges. Trop Med Health. 2025;53:77. 10.1186/s41182-025-00758-w 40448169 PMC12123987

[bib10] Gupta A, Sigdel TS. Integrating sustainable development goals in local plans: unlocking practices and challenges of local governments in Nepal. Heliyon. 2024;10:e39615. 10.1016/j.heliyon.2024.e39615 39497955 PMC11532878

[bib11] Gupta N, Gkrania-Klotsas E, Drexler J F et al. Nipah virus in south asia: from emergence to enduring preparedness' challenges. Clin Microbiol Infect. 2026. 10.1016/j.cmi.2026.03.021 41871738

[bib12] Haque Z, Adan MMIY, Zulfiker M S et al. Bifurcations and optimal control in Nipah virus epidemiology. PLoS One. 2026;21:e0342764. 10.1371/journal.pone.0342764 41811821 PMC12978506

[bib13] Hauser N, Gushiken A C, Narayanan S et al. Evolution of Nipah virus infection: past, present, and future considerations. TropicalMed. 2021;6:24. 10.3390/tropicalmed6010024 PMC8005932 33672796

[bib14] Joshi J, Shah Y, Pandey K et al. Possible high risk of transmission of the nipah virus in South and South East Asia: a review. Trop Med Health. 2023;51:44. 10.1186/s41182-023-00535-7 37559114 PMC10413696

[bib15] Khan S, Akbar SMF, Mahtab M A et al. Twenty-five years of Nipah outbreaks in Southeast Asia: a persistent threat to global health. IJID Reg. 2024;13:100434. 10.1016/j.ijregi.2024.100434 39308784 PMC11414670

[bib16] Khanal S, Schubert T, Boeckmann M et al. Assessing health facility preparedness in Nepal for addressing climate-related disasters and climate-sensitive diseases. Front Clim. 2025;7:1625829 10.3389/fclim.2025.1625829.

[bib17] Li H, Kim J V, Pickering BS. Henipavirus zoonosis: outbreaks, animal hosts and potential new emergence. Front Microbiol. 2023;14:1167085. 10.3389/fmicb.2023.1167085 37529329 PMC10387552

[bib18] Luby S P, Hossain M J, Gurley E S et al. Recurrent zoonotic transmission of Nipah virus into humans, Bangladesh, 2001–2007. Emerg Infect Dis. 2009;15:1229–35. 10.3201/eid1508.081237 19751584 PMC2815955

[bib19] Mehta R, Kushwaha A, Srivastava S et al. Dengue: a re-emerging infectious disease. Lessons from Nepal. Clin Infect Pract. 2026:100539 10.1016/j.clinpr.2026.100539.

[bib20] Mohandas S, Patil D, Mathapati B et al. Nipah virus survey in *Pteropus medius* of Eastern and Northeastern region of India, 2022–2023. Front Microbiol. 2024;15:1493428. 10.3389/fmicb.2024.1493428 39777153 PMC11703920

[bib21] Mohapatra P, Nazli Khatib M, Shabil M et al. Addressing the Nipah virus threat: a call for global vigilance and coordinated action. Clin Infect Pract. 2024;24:100390 10.1016/j.clinpr.2024.100390.

[bib22] Naidoo T, Morgenstern C, Doohan P et al. A systematic review of Nipah virus disease epidemiological parameters, outbreaks and mathematical models. medRxiv. 2026. 10.64898/2026.03.19.26348815 42447883

[bib23] Napit R, Khadka B, Khadka B et al. Integrated one health surveillance of zoonotic diseases at the wildlife-human interface: how pathogen detection informs spillover risks and transmission dynamics. medRxiv. 2025. 10.1101/2025.04.21.25326139

[bib24] Rayamajhee B, Pokhrel A, Syangtan G et al. How well the government of nepal is responding to covid-19? An experience from a resource-limited country to confront unprecedented pandemic. Front Public Health. 2021;9:597808. 10.3389/fpubh.2021.597808 33681124 PMC7925847

[bib25] Shah Y, Dumre S P, Joshi P et al. Nipah virus: a regional threat south Asia keeps underestimating. Lancet. 2026;407:1139. 10.1016/s0140-6736(26)00450-2 41831473

[bib26] Sharma J, Aryal A, Thapa GK. Envisioning a high-quality health system in Nepal: if not now, when?. Lancet Glob Health. 2018;6:e1146–e8. 10.1016/s2214-109x(18)30322-x 30196097 PMC7771520

[bib28] WHO. Nipah Virus Fact Sheet. Kathmandu, Nepal: World Health Organization, 2026. https://www.who.int/news-room/fact-sheets/detail/nipah-virus. (30 January 2026, date last accessed).

[bib29] Yadav P D, Baid K, Patil D Y et al. A One Health approach to understanding and managing Nipah virus outbreaks. Nat Microbiol. 2025;10:1272–81. 10.1038/s41564-025-02020-9 40437297

[bib30] Yeasmin D, Hossain M M, Haider S et al. The deadly drink: nipah virus transmission through date palm sap, cultural practices and the evolution of behavioral interventions in Bangladesh over two decades. J Infect Public Health. 2025;18:102949. 10.1016/j.jiph.2025.102949 40914133

